# Interface of Sleep Quality and Cognitive Health in Prevention of Dementia: SMRUTHI‐INDIA

**DOI:** 10.1002/alz70861_109025

**Published:** 2025-12-23

**Authors:** Shubham Gupta, Anu Gupta, Nilima N, Hina Narzari, Kritika Sharma, Aditi Dubey, Varuna Sharma, Sakshi Sharma, Kajal Fulara, Sneha, Kaamini Kashyap, Shikha Chaudhary, Krishan Kaushal, Phaniraj Vastrad, Priya darshanraj N, Sandip Bhattacharjee, Gajraj Singh Shekhawat, Harshath Ajay V, Sandesh J R, Ambika YV, Bishakha Sen, Shalina Jamatia, Priyanka Kumari Meena, Hitesh Tiwari, Shaily Bhushan, Sagar PM, Ripanjit Singh, Manish Acharjee, Kalpna Sharma, Aishwarya Bhovi, Mailarappa Padiyappa Hooli, Shilpa K, Naveen MR, Dipankar Deb, Pameli Jamatia, Pooja Sharma, Akhilesh Nagar, Tinku Yogi, Rishav Bhatia, Rishabh Gautam, MA Khan, Vaibhav Patil, Rajeev Aggarwal, Ashima Nehra, Subarna Roy, Manish Barvaliya, Subrata Baidya, Shampa Das, P K Anand, Sunil K Raina, Abhik Sinha, Venugopalan Y Vishnu, MV Padma Srivastava

**Affiliations:** ^1^ All India Institute of Medical Sciences, Delhi, Delhi India; ^2^ All India Institute of Medical Sciences, New Delhi, Delhi India; ^3^ All India Institute of Medical Sciences, New Delhi India; ^4^ All India Institute of Medical Sciences, New delhi, Delhi India; ^5^ MRHRU, Raichur, Karnataka India; ^6^ MRHRU, Khumulwmg, Tripura India; ^7^ Model Rural Health Research Unit (MRHRU), Jaipur, Rajasthan India; ^8^ Model Rural Health Research Unit (MRHRU), Sirwar, Raichur India; ^9^ Model Rural Health Research Unit (MRHRU), Sirwar, Raichur, Karnataka, India, Karnataka India; ^10^ Model Rural Health Research Unit (MRHRU), Khumulwng, Tripura India; ^11^ MRHRU, Jaipur, Rajasthan India; ^12^ Department of Community Medicine, Dr. RP Government Medical College, Tanda, Himachal Pradesh, India, Tanda, Himachal Pradesh India; ^13^ MRHRU, Sirwar, Karnataka India; ^14^ f) Department of Community Medicine, Dr. RP Government Medical College, Tanda, Himachal Pradesh, India, Una, Himachal Pradesh India; ^15^ Model Rural Health Research Unit (MRHRU), Raichur, Karnataka India; ^16^ MRHRU, Una, Himachal Pradesh India; ^17^ ICMR‐NITM, Belagavi, MRHRU Sirwar, Raichur, Karnataka India; ^18^ In charge MRHRU Khumulwmg, Tripura India; ^19^ Community Medicine AGMC, Khumulwng, Tripura India; ^20^ ICMR‐NIIRNCD, Jodhpur & Nodal Officer, MRHRU Bhanpur Kalan, Jaipur India; ^21^ Department of Community Medicine, Dr. RP Government Medical College, Tanda, Himachal Pradesh India; ^22^ ICMR Center for Ageing and Mental Health (CAMH), Kolkata India

## Abstract

**Background:**

SMRUTHI‐INDIA is a Cohort Multiple Randomized Controlled Trial (cmRCT) study examining risk and protective factors in preventing dementia. Globally, around 50 million adults are diagnosed with dementia, with India accounting for approximately 16% of these cases. Identifying protective factors, such as sleep quality, may help delay or prevent its onset. This study explores the potential protective role of sleep quality in maintaining cognitive health among the elderly.

**Method:**

This study employed a cmRCT design, allowing for repeated RCTs within an established cohort. The cohort was formed across four rural zones of India, focusing on dementia risk factors with annual neuropsychological assessments over five years. The current sample includes 2,402 individuals aged 55 and above. Sleep quality and cognition were assessed using the Pittsburgh Sleep Quality Index (PSQI) and Addenbrooke’s Cognitive Examination III (ACE‐III). Sex‐based comparisons were analysed using the Wilcoxon Rank‐Sum test.

**Result:**

Of 2,402 participants, 61.62% were females. Based on PSQI scores, 1,981 participants (83%) had good sleep quality (PSQI < 5), where data of 14 females and 10 males was not available due to lack of consent. Among females, 1,183 had good sleep quality; among males, 798 had good sleep quality. Cognitive performance, measured using ACE‐III, was compared across sleep quality groups using Wilcoxon Rank‐Sum test. In males, poorer sleep quality was significantly associated with lower cognitive scores (p = 0.01). However, no significant association was observed in females (p = 0.7), indicating a potential sex‐specific difference in the relationship between sleep quality and cognitive function.

**Conclusion:**

The findings of current study suggest that poor sleep quality is significantly associated with reduced cognitive performance in elderly males but not in females, highlighting a possible sex‐specific difference in how sleep affects cognitive health. These results underscore the importance of considering gender‐specific approaches when designing interventions to preserve cognitive function and potentially prevent dementia in aging populations. Further longitudinal analysis may help clarify the underlying mechanisms and long‐term impact of sleep quality on cognitive decline.

